# Supplementary material for: From sequence to enzyme mechanism using multi-label machine learning
Source: BMC Bioinformatics. 2014 May 19;15:150. doi: 10.1186/1471-2105-15-150 (PMC4229970; doi:10.1186/1471-2105-15-150)
Supplement: Additional file 2 — Java code of ml2db. Additional file ml2db_code.tar.gz contains the Java source code to run the multi-label machine learning experiments and save the results to database. The code’s Javadoc is included. [file 1471-2105-15-150-S2.zip › additional file 2/ml2db/ml2db/doc/main/ml2db/mulan/predict/class-use/PredictionsSaver.html]

Uses of Class main.ml2db.mulan.predict.PredictionsSaver


JavaScript is disabled on your browser.


- Overview
- Package
- Class
- Use
- Tree
- Deprecated
- Index
- Help

- Prev
- Next

- Frames
- No Frames

- All Classes

## Uses of Class main.ml2db.mulan.predict.PredictionsSaver

- Packages that use PredictionsSaver

  | Package | Description |
  | --- | --- |
  |  |  |
  | --- | --- |
  | main.ml2db.mulan.learn.evaluation |  |
  | main.ml2db.mulan.learn.traintest |  |
  | test.ml2db.mulan.predict |  |
- - ### Uses of PredictionsSaver in main.ml2db.mulan.learn.evaluation

    Methods in main.ml2db.mulan.learn.evaluation with parameters of type PredictionsSaver

    | Modifier and Type | Method and Description |
    | --- | --- |
    |  |  |
    | --- | --- |
    | `mulan.evaluation.Evaluation` | EvaluatorNoAUC.`evaluate(mulan.classifier.MultiLabelLearner learner, mulan.data.MultiLabelInstances data, boolean savePredictions, PredictionsSaver predSaver)` Evaluates a `MultiLabelLearner` on given test data set. |
    | `mulan.evaluation.Evaluation` | EvaluatorNoAUC.`evaluate(mulan.classifier.MultiLabelLearner learner, mulan.data.MultiLabelInstances data, java.util.List<mulan.evaluation.measure.Measure> measures, boolean savePredictions, PredictionsSaver predSaver)` Evaluates a `MultiLabelLearner` on given test data set using specified evaluation measures |
  - ### Uses of PredictionsSaver in main.ml2db.mulan.learn.traintest

    Methods in main.ml2db.mulan.learn.traintest that return PredictionsSaver

    | Modifier and Type | Method and Description |
    | --- | --- |
    |  |  |
    | --- | --- |
    | `PredictionsSaver` | TrainTestEvaluator.`getPredSaver()` |
  - ### Uses of PredictionsSaver in test.ml2db.mulan.predict

    Methods in test.ml2db.mulan.predict that return PredictionsSaver

    | Modifier and Type | Method and Description |
    | --- | --- |
    |  |  |
    | --- | --- |
    | `static PredictionsSaver` | PredictionsSaverTest.`getSaver(java.lang.String trainDatasetName, java.lang.String trainProps, java.lang.String testDatasetName, java.lang.String testProps, MulanLearner learner)` |
    | `static PredictionsSaver` | PredictionsSaverTest.`predictor1TrainOnTrain()` |

- Overview
- Package
- Class
- Use
- Tree
- Deprecated
- Index
- Help

- Prev
- Next

- Frames
- No Frames

- All Classes
